# A Report on the Condition and Organization of Local Societies

**Published:** 1863-09

**Authors:** J. Taft


### DENTAL REGISTER OF THE WEST.

VOL. XVII.] SEPTEMBER, 1863. [No. 9.

### Original Essays and Communications.

### A REPORT ON THE CONDITION AND ORGANIZATION OF LOCAL SOCIETIES.

Read before the American Dental Association.

BY J. TAFT.

At the last annual meeting of the American Dental Association, a committee, consisting of two members, was appointed for the purpose of conferring with the profession as opportunity might offer, in regard to the subject of local associations, especially in reference to the establishment of new organizations.

Associated effort and intercourse in all departments of human occupation, is regarded as a great element of power and efficiency. In the professions is this true, perhaps, to a greater extent than elsewhere, and in none more than in the dental profession. It, as a profession, is unlike all others; it is a blending of the principles of medicine with the mechanic arts, to such an extent and in such a manner as is nowhere else found: Hence it behooves him who would properly assume its duties, to acquaint himself with the general principles of medicine, not only theoretically, but practically to a considerable extent; and in addition to this there must be a high order of mechanical and artistic skill, and that of large range. This combination opens up a vast field, in the occupation of which associated effort comes in with immense power.

The question then occurs, why do not all avail themselves of the advantages thus obtainable?

And first, we apprehend that men generally in our profession, as elsewhere, have not been awake to the importance of the subject; most certainly this has been the case with the great majority of our professional brethren throughout the country, till within comparatively a recent period. Within the last few years, however, much has been done, especially in the United States, by associated effort; though it has wanted that system and arrangement that is desirable, or that would produce the largest results. It is owing, perhaps, to the existence of such efforts that the profession in this country occupies its present position.

Again, outside of large towns and cities, a want of contiguity has rendered it difficult for dentists to form associations, and convene often enough to keep an interest. This difficulty, however, is being surmounted by increased facility of communication. There is also a greater or more extended appreciation of the importance of the subject. Many who have seemed to be almost isolated, have attached themselves to permanent societies, and at very considerable distance from their field of labor.

The tendency now is to form dental associations at all feasible points; and we are happy to state the conviction that the formation of this National Association has been a great stimulus in that direction.

Again, jealousies and selfishness have kept many of the members of the profession asunder and isolated, when they should have been together.

The importance of associated effort was recognized and acted upon twenty years ago, the result of which was the organization of the American Dental Society. It was a pioneer, and did the work of a pioneer. It did not at any time influence any considerable portion of the profession, at least so far as members were concerned; though it had in its embrace many of the best men of that day in the profession.

It had for its object, "To promote union and harmony among all respectable and well-informed dental surgeons, to advance the science by free communication and interchange of sentiments, either written or verbal, between members of the Society; in fine, to give character and respectability to the profession, by establishing a line of distinction between the truly meritorious and skillful, and such as riot on the ill-gotten fruit of unblushing impudence and empiracism."

These were high and noble objects, and perhaps were carried out, so far as the influence of the Society extended. It did a good work, however, for its members in the days of its prosperity; but alas! evil days came upon it, when disputations, contentions and divisions came in and took away its prestige, usefulness, and ultimately its existence. Or perhaps we had better say it fulfilled its mission, did all it had the ability to do, and died a comfortable death. Its influence, however, did not then cease, for its records stand as a beacon-light, serving the double purpose of warning and directing. It numbered at one time about thirty members.

*The Mississippi Valley Association of Dental Surgeons*, was organized in the year 1844, by members of the profession from all parts of the Mississippi Valley. It is now the oldest dental society in existence. There have been, from the organization to the present time, over one hundred members. There are, at the present time, thirty-seven active members.

Its proceedings and operations are so well known to the profession, that any special details are unnecessary. Its meetings have been held annually, and always in the city of Cincinnati. It has secured the appellation of "The working Society." In addition to the production of essays, papers, discussions, etc., it has several times given premiums for teeth, instruments and appliances, and also gave a prize of one hundred dollars for a popular essay on dental surgery, which was published and circulated to a considerable extent by the profession in the West, and with the most happy results in every instance.

The officers at present are as follows, to-wit: E. Collins, President; H. R. Smith, Vice-President; H. A. Smith, Recording Secretary; Geo. F. Foote, Corresponding Secretary; J. Taft, Treasurer,—all of Cincinnati.

The delegates to the American Dental Association are:—Drs. J. P. Ullery, of Rising Sun, Ind.; H. McCullum, of Augusta, Ky.; E. Collins, George F. Foote, W. C. Duncan, of Cincinnati, O.; and W. H. Shadoan, of Withamsville, O.

From the year 1845 to 1855, there were a number of dental associations formed throughout the country; but few of which had any considerable duration. Some account of these might be to us interesting, had we more of their history.

*Pennsylvania Association of Dental Surgeons.*—This Society was organized in the city of Philadelphia, Pa., December 15th, 1845; and at first consisted of twenty-four members. "The time having arrived when it was expedient to organize an association of dental surgeons," a call, signed by most of the prominent members of the profession in that vicinity, was issued for a convention of dentists; this was held and resulted in the formation of a Society. A constitution and by-laws were framed and adopted.

The objects of the Association are set forth as follows; "To cultivate the science of dentistry, and all its collateral branches; to elevate and sustain the professional character of dentists; and to promote among them mutual improvement, social intercourse, and good feeling."

The constitution certainly was a model for that time, and indeed we could now scarcely suggest an improvement. The by-laws are elaborate, consisting of twenty-one articles; they are very minute in specifications, and very good.

This Society has gone steadily on, accomplishing a vast amount of good for the profession. Its transactions and discussions have usually been published, and were of much interest to the profession.

This Association now consists of fifty-three active, and six honorary members.

The present officers are—Dr. Wm. W. Fouche, President; Dr. J. F. Flagg, Vice-President; Dr. Geo. W. Ellis, Recording Secretary; Dr. C. A. Kingsbury, Corresponding Secretary; Dr. S. Dillingham, Treasurer; Dr. C. E. Hopkins, Librarian,

Permanent members of the American Dental Association: Drs. S. Dillingham, J. H. McQuillen, C. P. Fitch, Geo. T. Barker, J. F. Flagg, J. W. Van Osten, D. McFarlan, B. M. Gildea, J. L. Suesserotte, and Geo. W. Ellis.

The delegates are—T. L. Buckingham, C. N. Pierce, Charles Moore, and J. Hayhurst.

The Association meets once a month.

*The Michigan Dental Association*, was organized January 8th, 1856. It meets annually, on the third Monday of January, at such place as may be designated. There are eighteen active members.

The officers at present are as follows: H. Benedict, of Detroit, Mich., President; G. W. Stone, of Detroit, Vice-President; J. A. Watling, of Ypsilanti, Mich., Secretary; J. Mansfield, of Niles, Mich., Treasurer.

The names of the permanent members of the National Association are—Drs. Wm. Cahoon, and H. Benedict, of Detroit, Mich.; and J. A. Watling, of Ypsilanti, Mich.

The present dêlegates are—Drs. J. A. Robinson, A. F. Metcalf, C. E. Bartlett, and J. Mansfield.

This society is the only society in the State of Michigan, and may be considered a permanent institution.

*New York City Dental Association*, was organized on the 20th of March, 1860, with eleven members; during the first year, there were added eight new members. The meetings are held monthly, and the officers elected annually. At the meeting of organization the following Preamble was presented and adopted:

"WHEREAS, The rapid progress of the dental art in America can be clearly traced, in a great measure, to the interchange of thought elicited by the establishment of dental associations and dental periodicals; and *whereas*, the profession in other cities are organized and aiding in Dental progress, by associations and publications: It is therefore deemed expedient, for our own honor and interest, to organize a dental association in the city of New York."

Soon after the organization of the Society, the following resolution was adopted:

"*Resolved*, That the dental profession and the public be cordially invited to attend the meetings of this Society, with the exception of the regular quarterly meetings."

Thus the door of instruction was thrown open.

By reference to the proceedings, it will be seen that from the beginning the discussions have taken a wide range, embracing a great variety of subjects, all interesting and instructive to the true searcher for light.

The present officers are—T. H. Burras, President; W. B. Hurd, 1st Vice-President; W. H. Allen, 2d Vice-President; Charles D. Allen, Recording Secretary; N. W. Kingsley, Corresponding Secretary; A. A. Wheeler, Treasurer; Thos. Burgh, Librarian.

The delegates to this Association are—Drs. T. H. Burras, ......... Ambler, Thos. Burgh, Geo. Perrine, B. W. Franklin, John Allen, N. W. Kingsley, and O. A. Jarvis.

There are at present thirty-seven active members. This Society gives promise of permanency and great good.

*The Northern Ohio Dental Association*, was formed in Cleveland, March 6th, 1860, by five members originally; there are now nineteen. It meets annually, on the first Tuesday of May; though there are frequent called meetings.

The officers are—B. Strickland, of Cleveland, President;

H. P. Huntington, of Painesville, Vice-President; C. R. Butler, of Cleveland, Recording Secretary; B. F. Robinson, of Cleveland, Corresponding Secretary; F. S. Slosson, of Cleveland, Treasurer.

Drs. B. Strickland, C. Palmer, B. T. Spellman, and W. H. Atkinson are permanent members of the American Dental Association. The present delegates are Drs. C. R. Butler, J. C. Whinery, and A. E. Lyman.

*The Kentucky State Dental Association* was organized in April, 1860, and meets annually on the second Tuesday of July, at such place as may be appointed. There are twenty-one members in "good standing."

Drs. W. M. Rogers, W. D. Stone, and A. S. Talbert, are members of the American Dental Association.

The present Officers are—Dr. Richard Peckover, of Paris, President; Dr. J. A. McClelland, of Louisville, Vice-President; Dr. Stoddard Driggs, of Lexington, Secretary; Dr. W. G. Redman, of Louisville, Treasurer.

Delegates to the National Association:—Drs. W. G. Redman, Stoddard Driggs, J. A. McClelland, and H. Baldwin.

The Secretary, in speaking of the condition of the Society, says, "Our Society, just at the present time, can not be said to be very flourishing; but 'when this cruel war is over,' we expect to be found among the ardent and industrious." It is proper to observe here, that though this Society is located in a portion of our country that has been overrun by the scourge of war, yet it has not failed to hold its annual meetings, not only in name, but with spirit and interest.

*The Western Dental Society* was organized in 1856. Has forty members. Meets annually, at such places as the Association may select.

The officers are—Dr. H. E. Peebles, President; Dr. H. Barron, 1st Vice-President; Dr. A. Phillips, 2d Vice-President; Dr. C. W. Spalding, Recording Secretary and Treasurer; Dr. A. M. Leslie, Corresponding Secretary.

Delegate to the American Dental Association:—Dr. C. W. Spalding.

This Society has always been characterized by the unanimity and harmony of its meetings. It has accomplished much good for the profession in the West.

*The Brooklyn Dental Association* was organized on the 12th day of June, 1862; the membership at first consisting of eight persons. There are now forty active members. In speaking of the condition of the Society, the Secretary remarks: "Since its organization, the Association has held twenty-six meetings; a large majority of its members being present at each meeting. Thirty-five essays have been read before the Society by its members, each one reflecting credit upon its author. At every meeting, a subject or subjects pertaining to the profession have been discussed. Harmony and good feeling exist to that extent, that during the past year there has not been a jarring note among us."

The officers are—Dr. Wm. C. Parks, President; Dr. A. C. Hawes, Vice-President; Dr. Wm. B. Hurd, Secretary.

Last year there were no delegates from this to the National Association.

The present delegates are—Drs. A. A. Wheeler, C. A. Marvin, A. C. Hawes, J. C. Munroe, Wm. B. Hurd, A. W. Allen, G. A. Mills, and J. S. Latimer.

This Society is worthy of emulation, in many respects, and especially in the regularity, frequency and promptness of its meetings.

*The Cincinnati Dental Society* was formed September 8th, 1858, by the dentists of Cincinnati and vicinity. Its meetings were designed to be monthly; but owing to its proximity to the land of hostilities, and the excitement consequent thereupon, there have been two or three interruptions. Except these, meetings have been held two or three times in each month.

This Society numbers twenty members, nearly all of whom manifest much interest in the Society.

The officers are elected semi-annually, on the first Tuesday of September and March. At present they consist of the following persons, to-wit:

H. A. Smith, President; P. Knowlton, Vice-President; C. H. James, Secretary; H. R. Smith, Treasurer.

The following are permanent members from this Society to the National Association:—Drs. H. R. Smith and J. Taft.

The present delegates are—Drs. S. Wardel, J. G. Cameron, and B. D. Wheeler.

This Society has worked on, quietly and unostentatiously. It has recently published a journal for popular use, to be distributed gratuitously by the members to their patients. It is proposed to issue it quarterly.

It is interesting and important to the members of this Association to know the status and condition of all the organizations heretofore represented in this Body; but perhaps equally if not more interesting to obtain some indications of the future progress and career of this National Society.

We think there are some indications pointing in this direction in the institution of new Societies within the last year.

Since the last meeting of this Body, and notwithstanding the unfavorable circumstances arising from the condition of our country, five new organizations have been effected. Some of these, at least, were influenced to some degree in their formation by this Association. Members of the profession throughout the country begin to appreciate the fact that they can only enter this Association through local organizations, and that only in a representative capacity, or through this medium can they place themselves in the best possible position in the profession. In regard to the formation of three of these, your committee, in virtue of your appointment, were permitted to act *advisory* and *suggestive.* Your committee have to regret, however, that they have done far less than the importance of the matter demanded.

*The Western New York Dental Society* was organized in October, 1862, at Rochester, N. Y. There were, at the formation, thirty-eight members.

The following are the officers:—Dr. Charles W. Harvey, of Buffalo, President; Dr. E. F. Wilson, of Rochester, Vice-President; Dr. L. W. Bristol, of Lockport, Secretary; Dr. B. T. Whitney, of Buffalo, Treasurer.

It includes most of the prominent members of the profession in Western New York.

Two meetings are held annually on the first Tuesday in May and October, at such places as the Association may designate. There is active interest exhibited by the members; and doubtless the Association will go on to the full performance of its high trust.

The next organization in point of time is the *Pittsburg Dental Association* of *Western Pennsylvania.* It was formed in February last, with twenty-two members.

The officers are—Dr. James Orr, President; Dr. M. E. Gillespie, Vice-President: Dr. J. D. White, Secretary; Dr. J. Westby, Treasurer.

The delegates to the National Association are—Drs. Chas. Sill, J. Westby, James Orr, J. Vandervort, and J. S. King.

This Society has made an advance step in the establishment of a Dental *Institute* or *Infirmary*, for the treatment of the poor, as well as to subserve the interests of the profession.

This is a movement worthy of imitation wherever circumstances will admit, where members are so situated as to act efficiently. The objects that may be accomplished by such institutions are various and important. They may be the means of great good to those not able otherwise to obtain it.

Cases for *illustration*, *demonstration* and *experiment* may be thus procured. The members of the profession may here meet together, and compare, consult and aid each other, by suggestions upon actual cases that may be presented; in the treatment of diseases, and operations of all kinds.

Such institutions may be used with much efficiency against quackery,—indeed, this would be their legitimate tendency.

*The Central New York Dental Association* was next organized, on the 23d of last March, with twenty-nine members.

The officers for the year are—Dr. D. S. Ball, of Auburn, President; Dr. D. W. Perkins, of Baldwinsville, Vice-President: Dr. S. B. Palmer, of Tully, Recording Secretary; Dr. A. T. Smith, of Syracuse, Corresponding Secretary; Dr. E. M. Skinner, of Syracuse, Treasurer.

The Society has two regular meetings each year; the annual meeting on the second Tuesday of May, and the semi-annual on the second Tuesday of November.

*Odontographic Society of Pennsylvania.*—This Society was established in Philadelphia, May 19th, 1863, with the view of promoting professional and social intercourse among dental practitioners, and to encourage a disposition for investigation on their part in every direction which relates to the principles and practice of the profession or collateral sciences; with this object in view, essayists are regularly appointed to prepare dissertations embodying the results of actual experience, observation, research and reflection on such subjects; the papers being read at the monthly meetings, and are then subject matter for discussion on the part of the members.

The memberships are Active, Corresponding, and Honorary. The *active members* consisting of practitioners of dentistry residing in the State of Pennsylvania. The *corresponding members*, of practitioners of dentistry residing in other States of the Union, or in foreign countries, who manifest a disposition to advance the science and art of the profession by contributing to its literature. The *honorary members*, of practitioners of dentistry who have honorably retired from practice; or of medical practitioners or others who have made valuable contributions to the science or art of dentistry.

The *stated meetings* are held on the first Tuesday of each month of the year, at eight o'clock P. M. All the meetings, with the exception of the annual meeting, are devoted *exclusively* to the reading of essays and the discussion of subjects connected with the principles and practice of dentistry and collateral sciences. The recommendation and election of members, and donations to the library, are in order at these meetings, but no other business can be brought forward.

The *Annual meeting* in May, is appropriated to the election of officers, the financial concerns of the Society, receiving reports of committees, and, in general, to such business as does not appertain to the scientific transactions of the Society.

Special meetings may be convened by the President, or at the request of five members.

The *Transactions* of the Society, under the name of the ODONTOGRAPHIC SOCIETY of *Pennsylvania*, are printed under the supervision of the executive committee.

Newly-elected *active* members, who have paid their initiation fee and annual dues, are presented with the Transactions for the season in which they are elected, and are entitled to a copy each successive year on the payment of their annual dues; but no old member, whose dues are in arrears, or newly-elected member whose initiation fee and annual dues have not been paid, is entitled to the Transactions.

The Transactions are purchasable by all members of the Society, and others, at their prime cost; and are exchangeable for the Transactions of other Societies.

Although the Society was organized quite recently (within three months), the *active* members already number twenty-seven. The meetings have been regularly attended, and the papers read at them have elicited animated, interesting and instructive discussions. The indications are that the Society will be the means of great good to its members and others.

The officers are as follows:—Dr. Jacob Gilliams, President; Dr. John McCalla, 1st Vice-President; Dr. C. A. Kingsbury, 2d Vice-President; Dr. J. H. McQuillen, Corresponding Secretary; Dr. L. M. Louson, Recording Secretary; Dr. Thomas Wardle, Treasurer; Dr. Wm. P. Henry, Librarian; Drs. J. Foster Flagg, S. R. Scriven and Wm. Gorges, Executive Committee,

Delegates to the American Dental Association—Drs. Jacob Gilliams, John McCalla, C. A. Kingsbury, Wm. Gorges, Ambler Tees, and W. K. Brenizer.

*The Iowa State Dental Association* was organized in July, 1863; quite a number of the leading dentists of the State were present and participated in its proceedings.

In this hasty glance at some of the Dental Associations of our country, many and various thoughts and suggestions arise. There may, and doubtless will be, some diversity of opinion, as to the most profitable course to be pursued in the conduct of local Associations. The methods of operation will, to some extent, be modified by circumstances, such as the facility of meeting, the number of members, etc. In regard to the frequency of meeting, it will be seen that in those Associations upon which we have reported, there is quite a diversity,—some hold but one meeting annually; and on the other extreme, some meet once a week, and others at various intermediate periods. In regard to the frequency of meeting, each Society must determine what is best for itself.

In sparsely settled parts of the country, or where the members are scattered over a large scope of country, perhaps not more than from two to six meetings annually would be practicable; while in large cities, once a month, or even once a week, Societies may meet profitably. Always such frequency should be observed as would elicit the greatest interest.

The subject of open or public meetings, is one upon which there has been diversity, in practice at least. Some admit none but actual members, except upon special invitation; others make their meetings free to all their professional brethren who choose to meet with them. Oftentimes physicians are invited; and some make their meetings open to *all* who may desire to attend.

The exclusive feature savors rather too much of selfishness, to say the least; and it certainly does not result in the greatest good to the largest number,—which we think should be the criterion, in this matter.

Especially would it always be desirable and profitable to have the suggestions and opinions of physicians, and particularly in reference to many questions that arise in regard to those things with which they are more familiar. The ultimate efficiency of Dental Associations depends much upon their immediate and prospective permanency, as well as the promptness and uniformity of attendance by the members; a desire for improvement and progress should stimulate to a uniform promptness. But it may be proper to inquire, whether there may not be additional influences brought to bear, to bind men closer together, and to give greater assurance of permanency to such Institutions. We think such additional security exists in the possession of property. Hence we would have every local Society possessed of property, in some form; of course it should be of that character which should be most available in carrying out the objects of the Society. It would very properly consist of libraries, museums, collections of optical, chemical, philosophical, and mechanical instruments and appliances; indeed, everything that may be employed to aid in the development and advancement of our profession, both in a scientific and artistic aspect.

We would therefore suggest that every Society, as soon as practicable, carry out these suggestions, because of the advantage which is directly derivable therefrom, and also, because character and permanency will thereby be secured.

Another matter to which all Associations should direct their attention, is popular dental education. This is a subject of vastly more importance than is usually attached to it. We do not propose here to discuss its advantages, nor to specify particulars in carrying out the method; but we will suggest, and indeed urge, that every Society should immediately set about some systematic and efficient method of giving information to the people; the work has already been commenced, though not extensively.

As to how it should be done, various suggestions may arise. Some have issued essays, others are publishing journals; and in many cases, individuals have published fugitive pieces in the current prints of the day.

The subject of Dental Hospitals is one that has elicited some thought and attention.

Our English brethren, some three years ago, established an institution of this kind, from which they hoped much. It is still in operation, but with what measure of success, and what is its present condition, we are not advised.

The "Pittsburg Dental Association" is making an experiment in this direction, and we believe the first in this country. It is to be hoped that other Societies will direct their attention and effort in the same direction.

It is a very gratifying indication, that the profession seems to be reaching out in *new* channels for instrumentalities that shall aid in its progress and development, as well as working in the old.

Though the dental profession has been hitherto marching, with rapid strides, up to that point where it takes rank and position, fresh and vigorous, with other and older professions, yet a more glorious day seems to be dawning upon it, in which more rapid progress shall be made, when all the resources treasured in nature's storehouse shall be brought forth, and applied. This is our work.

